# Use of intraoperative ultrasound in a benign epidermoid cyst-like tumor lesion with testicular preservation: Case report

**DOI:** 10.1016/j.radcr.2025.06.010

**Published:** 2025-07-16

**Authors:** Katherine Márquez-Bayona, Daniel Felipe Gomez-Cadena, Luz Angela Moreno-Gomez, Camilo Alberto Orjuela-Rodriguez, Jennifer Richardson-Maturana

**Affiliations:** a Department of Pediatric Radiology, HOMI Fundación Hospital Pediátrico la Misericordia, Carrera 14 # 1-65, Bogotá, Colombia/Universidad Nacional de Colombia, Bogotá, Colombia; b Department of Pediatric Radiology, HOMI Fundación Hospital Pediátrico la Misericordia, Carrera 14 # 1-65, Bogotá, Colombia/Universidad de los Andes, Bogotá, Colombia

**Keywords:** Epidermoid cyst, Ultrasound, Testicular tumors, Intraoperative ultrasound

## Abstract

Epidermoid cysts are rare benign lesions in adolescence, commonly presenting as slow-growing, nonpalpable masses lined with squamous epithelium, filled with keratinized material. Testicular ultrasound is the study of choice for the diagnosis of these lesions, and its intraoperative use has been shown to be beneficial for the patient's hormonal and reproductive prognosis. In this study, we present the case of a 17-year-old adolescent male who came to the urology office with a sensation of mass in the right testicle with negative tumor markers. Tumor resection was performed with intraoperative ultrasound guidance and freezing biopsy, with confirmation of the diagnosis of squamous cell cyst, which allowed the decision to preserve the testicle. Epidermoid cysts are benign testicular lesions that commonly do not require orchiectomy. The correct imaging identification with pre- and intraoperative ultrasound is acquiring an important role in the management of these cases by allowing testicular preservation decisions, which offers a better hormonal and reproductive prognosis for patients.

## Introduction

Epidermoid cysts are benign lesions characterized by a lining of squamous epithelium, filled with keratinized material, slow growing with components inside and outside the skin [1,2]. Ultrasonographically they are described as lesions averaging 3 cm in diameter, with characteristic onion layered image caused by alternating hypo- and hyperechogenic images within, although they are also often described as a bull's eye image [3].

With the increasing use of ultrasound, the importance of intraoperative ultrasound in determining the position and depth of the lesion, assessment of ischemia and vasculature, and confirmation of complete excision of the mass has been seen [4].

## Case report

A 17-year-old adolescent male presented to the urology outpatient clinic with a nonpainful sensation of a mass in the right testicle, not associated with urinary symptoms, fever, weight loss or any other symptomatology. The patient had no important pathological history, and physical examination revealed induration in the upper third of the right testicle with no palpable nodule.

The initial out-of-institutional ultrasonography reports a hypoechoic intratesticular nodule in the right testicle with lobulated borders and calcified wall, with scarce vascularization on color Doppler. It also has tumor markers such as alpha-fetoprotein, lactate dehydrogenase, and negative beta-subunit human chorionic hormone. Due to these characteristics, it was proposed to prioritize scrotal exploration with freezing biopsy with intraoperative ultrasound support, seeking to guide behavior towards preserving surgery or orchiectomy according to the pathology results.

**Fig. 1:**
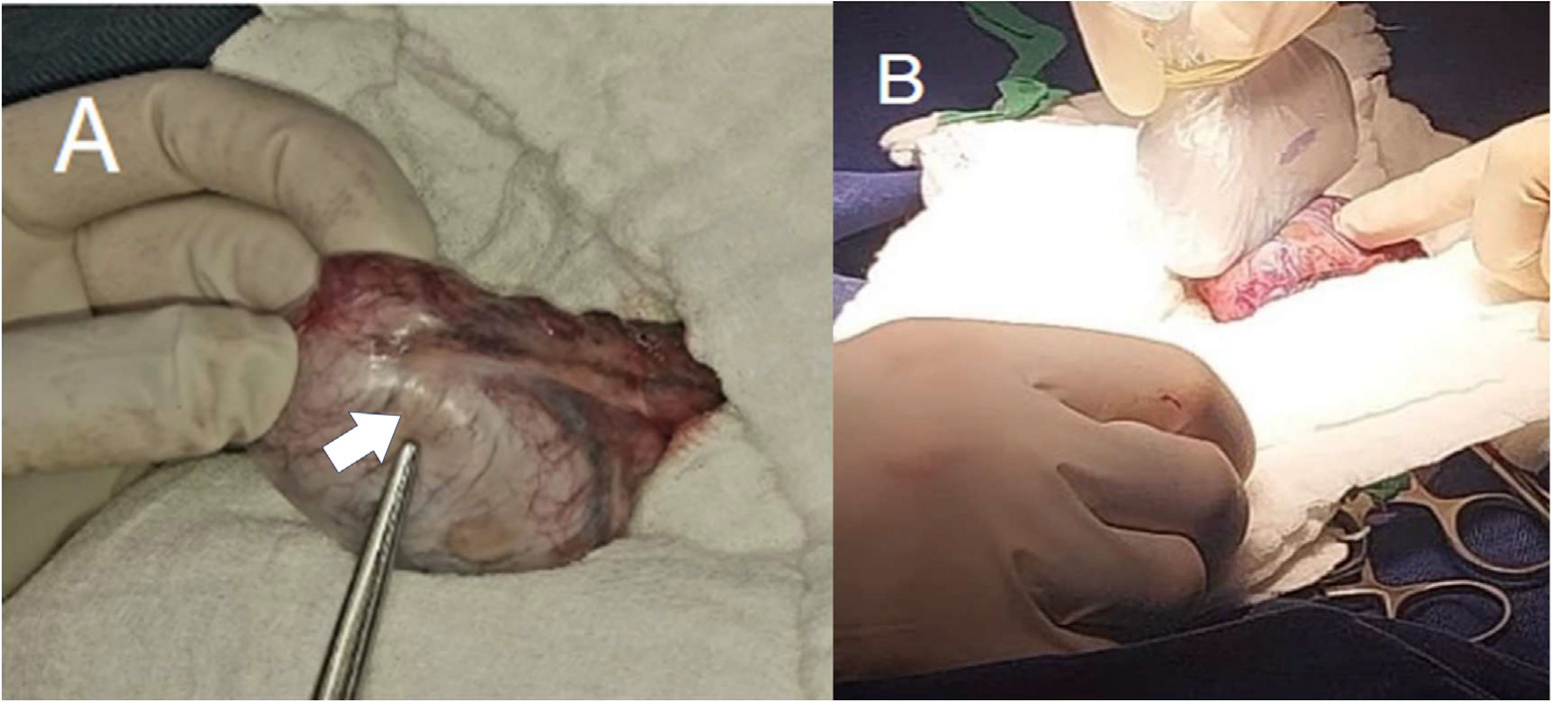
(A) Macroscopic identification of the lesion (Arrow). (B) Intraoperative ultrasound.

**Fig. 2:**
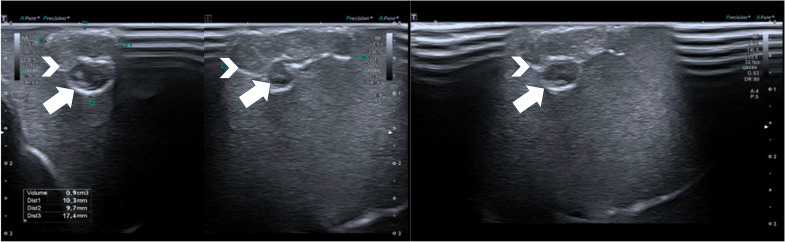
Presurgical intraoperative ultrasound. Right testicular intraparenchymal lesion (arrow), adjacent to the tunica albuginea at its anterior margin (arrow head), hypoechoic, polylobulated, circumscribed with peripheral echogenicity, with slight peripheral flow on color Doppler.

**Fig. 3:**
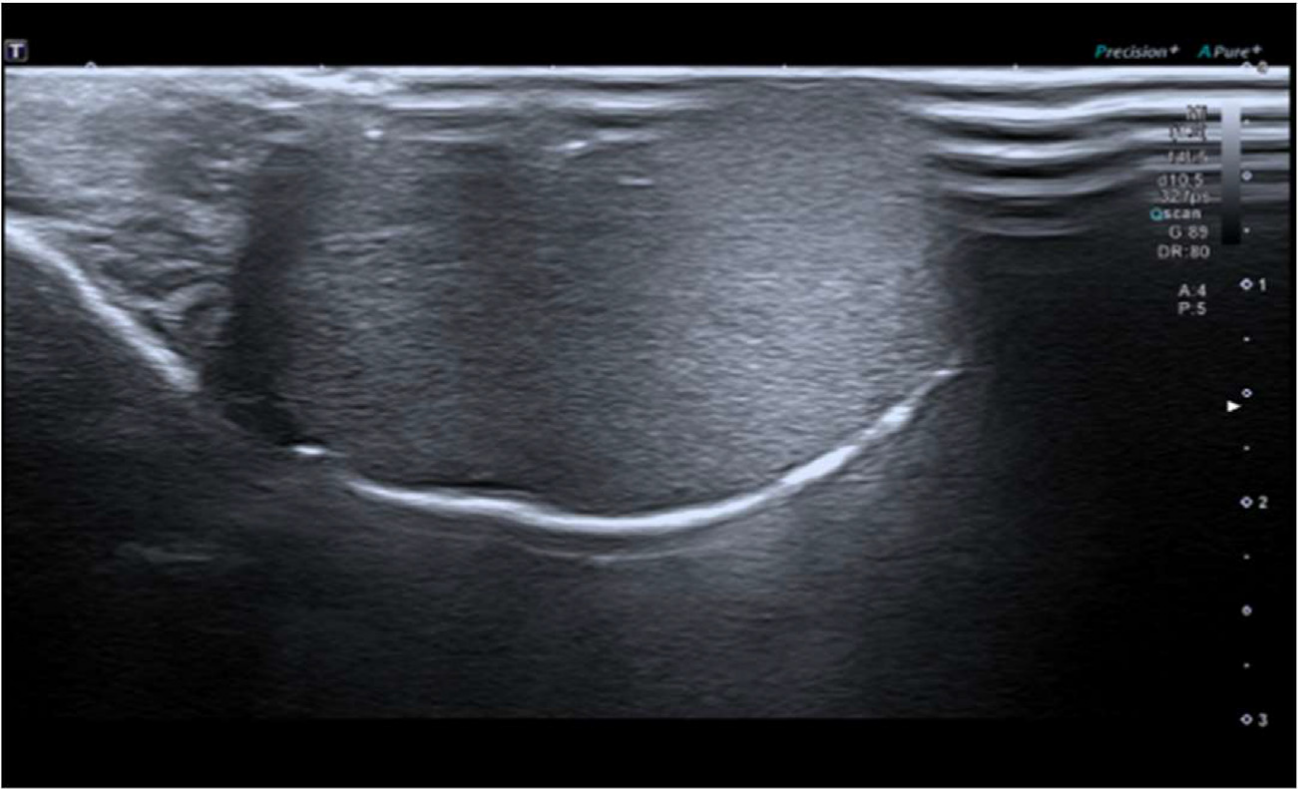
After surgery, no residue is observed by this imaging technique, which is confirmed by freeze biopsy.

During the surgical procedure, with exteriorization of the right testicle via inguinal route, complete excision of the lesion is performed by means of intraoperative ultrasound guidance performed by a pediatric radiologist (Fig. 1), which allows to determine the exact location of the intraparenchymal testicular lesion, adjacent to the tunica albuginea in its anterior margin, hypoechoic center, multilobulated, circumscribed, with peripheral echogenicity (target image), with slight peripheral flow to the Doppler-color analysis and previously known measures (Fig. 2). Ultrasound control of the surgical site was performed without finding tumor residue. On the other hand, the freezing biopsy showed an epidermoid cyst, so an intraoperative decision was made to perform testicular preservation (Fig. 3).

Finally, the third postoperative day was followed up with an official pathology report describing the finding of a lesion with a dense fibroconnective wall with calcified areas, nonvisible epithelium and in the external wall scarce usual testicular tissue, with material of a horny scale type in its lumen and bed with testicular parenchyma without evidence of atypia. The postoperative evolution was satisfactory.

## Discussion

Testicular tumors are infrequent neoplasms in male minors, accounting for about 2% of childhood tumors, with 2 peaks of presentation: in the neonatal stage and adolescence [5]. The most common testicular tumors are teratomas and yolk sac tumors, with 50% and 15% of cases, respectively; followed by epidermoid cysts with 15% and stromal tumors with 10% [5]. Epidermoid cysts are defined as benign lesions with a coating of squamous epithelium, filled with keratinized material, slow growing with components inside and outside the skin [1,2], have a mesothelial origin, originate mainly in the testicular parenchyma or tunica albuginea and mainly affect the epididymis [6]. Their most common location is intratesticular and they are usually misdiagnosed as malignant lesions [7,8].

Diagnostic imaging is of vital importance for the management of these neoplasms, with ultrasound and MRI being the most commonly used modalities for the evaluation of testicular pathology [9]. These allow determining the location and characteristics of the lesions, which are important for the differentiation between benign and malignant lesions, as well as for their management and postoperative follow-up (contralateral or distant recurrence and testicular volume) [9]. In this regard, a high-resolution ultrasound study, between 7.5 and 12.5 MHz, grayscale and color Doppler is considered the initial standard for the diagnosis of testicular masses [9]. Additionally, the use of other complementary modalities such as contrast-enhanced ultrasound and elastography has been described to improve the differentiation between malignant and benign lesions [9].

Case series seeking to describe the characteristics of epidermoid cysts in pediatric patients have been reported in the literature. They have been described as single, unilateral masses, with an average size of 2 to 3 cm in diameter that can be described as a well-defined, circumscribed, intratesticular lesion with normal surrounding parenchyma [10]. They commonly present a hypoechoic ring, with heterogeneous content with an echogenic center alternating between hypo- and hyperechogenic layers (which are made up of keratin layers) [3,11], described as a bull's eye or onion skin lesion (depending on the distribution of these layers) [12].

In the same way, the use of intraoperative ultrasound in pediatric surgery is growing, where it has shown to be useful not only in preoperative diagnosis but also in guiding surgical resection and allows better decision-making during surgery, especially in reducing surgical time and improving the outcome for the patient [13]. Regarding testicular tumors, the importance of intraoperative ultrasound lies in marking the position prior to the approach, determining the position and depth of the lesion, and, at the end of the excision of the lesion, evaluating the ischemia and vasculature, and confirming the complete removal of the lesion [4]. In pediatric urology, intraoperative ultrasound has shown great importance in preserving testicular tissue due to the importance of its reproductive and hormonal function [14]. However, it must be taken into account that, despite its clear advantages, one of its limitations can be operator dependence, being necessary the correct training to ensure an adequate technique [15].

## Conclusion

Testicular epidermoid cysts are benign tumors of infrequent presentation in adolescence, but they are commonly mistaken for malignant tumors that are usually led to partial or total orchiectomies, with important repercussions in their hormonal and reproductive development. Due to its easy access and low cost, ultrasound is the study of choice for the identification of these masses; and its intraoperative use has shown to reduce the need for radical management, preserving testicular tissue and offering a better outcome for patients. It should be emphasized that it has the limitation of being operator dependent, but with that arises the need to increase the use and training in intraoperative ultrasound to improve postoperative outcomes of patients.

## Ethical considerations

Taking into account the regulations governing medical research in Colombia, and in accordance with the stipulations of resolution 8430 of 1993, it is considered a research without risk since it is a case report through the review of clinical history and images, and it does not make a change in the patient's behavior or treatment. Regarding the stipulations of the personal data protection law, the corresponding informed consent was signed by the patient's legal representative (mother), with prior clarification of the purpose of the study, obtaining her express written acceptance to use the clinical history and the data included in it, emphasizing the main use of medical and imaging data, and that access to them is limited to the authors of the study. In addition, it is clarified that the research follows the guidelines of international standards, and that, during the writing of the manuscript and subsequent oral or graphic presentations, no sensitive data that would allow patient identification will be given.

## Patient consent

Taking into account the regulations governing medical research in Colombia, and in accordance with the stipulations of resolution 8430 of 1993, it is considered a research without risk since it is a case report through the review of clinical history and images, and it does not make a change in the patient's behavior or treatment. Regarding the stipulations of the personal data protection law, the corresponding informed consent was signed by the patient's legal representative (mother), with prior clarification of the purpose of the study, obtaining her express written acceptance to use the clinical history and the data included in it, emphasizing the main use of medical and imaging data, and that access to them is limited to the authors of the study. In addition, it is clarified that the research follows the guidelines of international standards, and that, during the writing of the manuscript and subsequent oral or graphic presentations, no sensitive data that would allow patient identification will be given.

## References

[1] Treadwell P. (2021). Epidermal inclusion cyst. Atlas Adolesc Dermatol.

[2] Fujino J., Yamamoto H., Kisaki Y., Ishimaru Y., Uchida H., Mori Y. (2004). Epidermoid cyst: rare testicular tumor in children. Pediat Radiol.

[3] Martinez Rios Arellano C., Kozakewich H.P., Diamond D., Chow J.S. (2011). Testicular epidermoid cysts in children: sonographic characteristics with pathological correlation. Pediat Radiol.

[4] Bertolotto M., Pavan N., Valentino M., Liguori G., Bucci S., Barozzi L. (2017). The role of intraoperative ultrasound for testicular masses. Atlas Ultrason Urol Androl Nephrol.

[5] Sangüesa C., Veiga D., Llavador M., Serrano A. (2020). Testicular tumours in children: an approach to diagnosis and management with pathologic correlation. Insight Imag.

[6] Ayala-Samaniego L.H., Merino-Zumba J.D., Pérez-Bravo T.E., Morejón-Alarcón J.E. (2022). Quiste epidermoide testicular en la edad pediátrica. Polo del Conocimiento.

[7] Ahmed A., Ridhorkar V., Goel D., Suryawanshi A. (2023). Unmasking the uncommon: a case report of scrotal epidermoid cysts in a nine-year-old boy. Cureus.

[8] Usui K., Yamashita R., Sakura Y., Nakamura M., Shinsaka H., Matsuzaki M., Niwakawa M. (2024). Epidermoid cyst of the testis: A report of three cases. Clinical case reports.

[9] Hermann A.L., L’Herminé-Coulomb A., Irtan S., Audry G., Cardoen L., Brisse H.J. (2022). Imaging of pediatric testicular and para-testicular tumors: a pictural review. Cancers.

[10] Ghazle H., Apeland T. (2019). Epidermoid cyst of the testis: sonographic characteristic appearance. J Diagnost Med Sonography.

[11] Cho J.H., Chang J.C., Park B.H., Lee J.G., Son C.H. (2002). Sonographic and MR imaging findings of testicular epidermoid cysts. Am J Roentgenol.

[12] Ateş F., Kara T., Durmaz M.S., Bayraktar A.M., Gurcan T. (2018). Case of testicular epidermoid cyst: sonographic and histopathologic findings. J Surg Med.

[13] Bawazir O., Bawazeer O.A. (2021). Ultrasound in pediatric surgery; intraoperative applications of the growing technology. Ann Pediat Surg.

[14] Appleyard W., Meshaka R., Bebi C., Cho A., Chowdhury T., Smeulders N. (2024). Intraoperative ultrasound-guided paediatric urological surgery: a pictorial review. Pediat Radiol.

[15] Ukimura O., Okihara K., Kamoi K., Naya Y., Ochiai A., Miki T. (2008). Intraoperative ultrasonography in an era of minimally invasive urology. Int J Urol.

